# Estimated allele substitution effects underlying genomic evaluation models depend on the scaling of allele counts

**DOI:** 10.1186/s12711-017-0355-9

**Published:** 2017-10-30

**Authors:** Aniek C. Bouwman, Ben J. Hayes, Mario P. L. Calus

**Affiliations:** 1 Animal Breeding and Genomics Centre, Wageningen Livestock Research, P.O. Box 338, 6700 AH Wageningen, The Netherlands; 2 Queensland Alliance for Agriculture and Food Innovation, Centre for Animal Science, University of Queensland, Brisbane, QLD Australia; 3 Department of Economic Development, Jobs, Transport and Resources, Government of Victoria, 5 Ring Rd., Bundoora, VIC 3083 Australia

## Abstract

**Background:**

Genomic evaluation is used to predict direct genomic values (DGV) for selection candidates in breeding programs, but also to estimate allele substitution effects (ASE) of single nucleotide polymorphisms (SNPs). Scaling of allele counts influences the estimated ASE, because scaling of allele counts results in less shrinkage towards the mean for low minor allele frequency (MAF) variants. Scaling may become relevant for estimating ASE as more low MAF variants will be used in genomic evaluations. We show the impact of scaling on estimates of ASE using real data and a theoretical framework, and in terms of power, model fit and predictive performance.

**Results:**

In a dairy cattle dataset with 630 K SNP genotypes, the correlation between DGV for stature from a random regression model using centered allele counts (RRc) and centered and scaled allele counts (RRcs) was 0.9988, whereas the overall correlation between ASE using RRc and RRcs was 0.27. The main difference in ASE between both methods was found for SNPs with a MAF lower than 0.01. Both the ratio (ASE from RRcs/ASE from RRc) and the regression coefficient (regression of ASE from RRcs on ASE from RRc) were much higher than 1 for low MAF SNPs. Derived equations showed that scenarios with a high heritability, a large number of individuals and a small number of variants have lower ratios between ASE from RRc and RRcs. We also investigated the optimal scaling parameter [from − 1 (RRcs) to 0 (RRc) in steps of 0.1] in the bovine stature dataset. We found that the log-likelihood was maximized with a scaling parameter of − 0.8, while the mean squared error of prediction was minimized with a scaling parameter of − 1, i.e., RRcs.

**Conclusions:**

Large differences in estimated ASE were observed for low MAF SNPs when allele counts were scaled or not scaled because there is less shrinkage towards the mean for scaled allele counts. We derived a theoretical framework that shows that the difference in ASE due to shrinkage is heavily influenced by the power of the data. Increasing the power results in smaller differences in ASE whether allele counts are scaled or not.

## Background

Genomic evaluation is used to predict direct genomic values (DGV) for selection candidates in breeding programs. In addition to the DGV, allele substitution effects (ASE) are or can be computed using genomic evaluation models. An ASE represents the effect that the presence of a copy of that allele has on the phenotype. This also applies for the estimation of such effects in genomic evaluation. The loci used do not have to be the causal variants; if they are in linkage disequilibrium (LD) with the causal loci, they can pick up the correlated part of the ASE of the causal loci. The estimated ASE from genomic evaluations can be used for various additional purposes such as rapid computation of DGV for newly genotyped individuals by multiplying their allele counts with the ASE [1], in genome-wide association studies (GWAS) to get insight on the genetic architecture of a trait [2, 3], and to estimate DGV based on small genomic regions, so-called ‘local DGV’ for quantitative trait loci mapping [4, 5].

Several genomic evaluation models estimate ASE first to predict the DGV, e.g., Bayesian stochastic search variable selection, SNP-best linear unbiased prediction (BLUP) or ridge regression [6, 7]. Other methods such as genomic (G)BLUP, genomic restricted maximum likelihood estimation (GREML), and one-step methods, use a genomic relationship matrix (GRM) that is constructed from the SNP genotypes [8–10]. The DGV are then predicted as a result of solving the mixed model equations, and ASE are not explicitly computed. However, with a GBLUP or GREML approach, it is straightforward to back-solve the ASE from DGV based on the genotypes of the animals [11].

All genomic evaluation models require genotypes that are either used directly or to construct a GRM. There are different genotype coding methods for the three possible genotypes: homozygous allele 1 (e.g., *AA*), heterozygous (e.g., *AB*) and homozygous allele 2 (e.g., *BB*). Often the genotypes are represented as the number of copies of one allele (e.g., counting the *B* allele in the above example: 0, 1, 2), which means that, in genomic evaluation models, the ASE is estimated for the allele that is being counted. These allele counts can be centered resulting in a mean of 0, or both centered and scaled resulting in a mean of 0 and a standard deviation of 1. Using centering only in genomic evaluation gives ASE directly, however, using centered and scaled allele counts results in estimated effects for the scaled genotypes, instead of for 0, 1, 2 genotypes, and an additional transformation is needed to obtain the actual ASE. Stranden and Christensen [12] showed that differences in genotype coding gave correlations between ASE close to 1 (higher than 0.9998) and the same DGV as long as the estimated general mean was included in the DGV. However, they looked at centering and did not include scaling in the genotype coding methods studied. As indicated by de los Campos et al. [13], centering only influences the intercept, but scaling results in less shrinkage towards the mean for low minor allele frequency (MAF) variants compared with variants with intermediate MAF.

In the past, low MAF variants were often ignored by applying a MAF cut-off of 1 to 5%, or a minimum number of copies of the minor allele present in the population, because such variants were considered unreliable [14]. Moreover, SNP genotyping platforms in livestock species have been developed such that mainly common SNPs are on the SNP-panel of commercial genotyping chips. However, with the recent rise in available whole-genome sequence data, the use of rare variants in genomic evaluation and GWAS is increasing.

Goddard [15] indicated that optimal long-term genomic selection is achieved by putting more emphasis on SNPs with a low frequency of the favorable allele, such that all SNPs will be fixed at the same moment in time. Jannink [16] also showed that putting more weight on favorable alleles with a low frequency benefits long-term selection since the final gain from weighted genomic selection is higher. In conservation genetics, it might be desirable to put more emphasis on rare alleles to preserve the alleles that are at high risk of disappearing in a few generations. Eynard et al. [17] showed that relationships between individuals based on variants with a MAF between 1 and 5% are significantly different from relationships based on more common variants, and concluded that for conservation of rare alleles the relationships should be estimated using scaled allele counts.

Given that the use of low MAF variants in genomic evaluation is likely to increase, scaling may become a more important consideration for the estimation of ASE. This paper shows the impact of scaling the centered allele counts on the estimation of ASE. An SNP-BLUP model was applied to dairy cattle data to estimate DGV and ASE, using different scaling parameters. We present a theoretical framework to show the origin of the difference in ASE, resulting from scaling, and the impact of the power of the data on this difference. In addition, the best-fitting scaling parameter and that with the best predictive performance were investigated.

## Methods

### Data

**Fig. 1:**
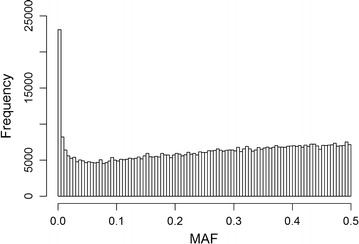
Histogram of minor allele frequencies (MAF) of single-nucleotide polymorphisms

Daughter yield deviations (DYD) for stature from 5554 Holstein bulls were available from CRV (Cooperative Cattle Improvement Organization, Arnhem, The Netherlands), as well as the number of daughters used to estimate the DYD (on average 549 daughters). The bulls were genotyped with the Illumina BovineHD Bead chip (734,403 SNPs; Illumina Inc., San Diego, CA, USA), or genotyped with a 50 K SNP panel and imputed to high-density (HD). SNPs with less than five copies of the minor allele segregating in the population were discarded. In addition, each possible SNP genotype had to occur at least once (i.e., at least one heterozygote and one homozygote carrying the minor allele), resulting in a final set of 627,440 SNPs. MAF ranged from 0.00045 (i.e., five alleles present in the population) to 0.5 with an average of 0.22; the frequency distribution of MAF is shown in Fig. 1.

### Genomic evaluation

To show the impact of scaling on ASE, the effects were estimated with a random regression model (SNP-BLUP) using two different genotype coding methods: centered allele counts (RRc) and centered and scaled allele counts (RRcs). The SNP-BLUP model is a random regression model, which estimates the regression coefficients with BLUP, assuming a normal distribution. The following SNP-BLUP models were solved using ASReml software [18]:

Centered (RRc):

$${\mathbf{y}} = \mathbf{1}\mu + {\mathbf{Zb}}_{{{\mathbf{RRc}}}} + {\mathbf{e}},$$

where ${\mathbf{y}}$ is a vector with phenotypes, here stature DYD; $\mu$ is the intercept; ${\mathbf{Z}}$ is a $n \times N$ design matrix containing centered allele counts for all individuals, where $n$ is the number of animals and $N$ the number of SNP; ${\mathbf{b}}_{{{\mathbf{RRc}}}}$ is a vector of random unknown SNP effects, the SNP effects were assumed to be identically and independently distributed with a mean 0 and variance $\sigma_{g}^{2}$, i.e., ${\mathbf{b}}\sim N\left( {0,{\mathbf{I}}\sigma_{g}^{2} } \right)$; and ${\mathbf{e}}$ is a vector of residual errors. The model assumed that ${\mathbf{e}}\sim N\left( {0,{\mathbf{D}}\sigma_{e}^{2} } \right)$, where ${\mathbf{D}}$ is a diagonal matrix with elements computed as $\frac{1}{{wt_{i} }}$, with weight $wt_{i}$ being the number of daughters of individual $i$ on which the DYD of $i$ was based, and $\sigma_{e}^{2}$ is the residual variance. Elements of ${\mathbf{Z}}$ are computed as $z_{ij} = x_{ij} - 2p_{j}$, where $x_{ij}$ is an element of the ${\mathbf{X}}$ matrix containing the SNP genotype for individual $i$ at locus $j$ coded as 0, 1, or 2; and $p_{j}$ is the frequency of the allele whose homozygous genotype is coded as 2 at locus $j$. Note that $2p_{j}$ is the mean allele count of the SNP used for the centering, and that the estimated SNP effects ${\hat{\mathbf{b}}}_{\text{RRc}}$ are the estimated ASE, i.e., ${\hat{\boldsymbol{\upalpha }}}_{\text{RRc}} = {\hat{\mathbf{b}}}_{\text{RRc}}$.

Centered and scaled (RRcs):

$${\mathbf{y}} = \mathbf{1}\mu + {\mathbf{Wb}}_{{{\mathbf{RRcs}}}} + {\mathbf{e}}.$$

For centered and scaled allele counts, the ${\mathbf{Z}}$ matrix was replaced by a ${\mathbf{W}}$ matrix, which contained elements computed as $w_{ij} = \frac{{\left( {x_{ij} - 2p_{j} } \right)}}{{\sqrt {2p_{j} \left( {1 - p_{j} } \right)} }}$. Note that $2p_{j}$ is the mean allele count of the SNP used for the centering, and that $\sqrt {2p_{j} \left( {1 - p_{j} } \right)}$ is the standard deviation used for the scaling. Since ${\mathbf{W}}$ contains scaled allele counts, the estimated SNP effects are not ASE, i.e., they are not equal to half the difference of the value between the two homozygotes [19]. The ASE can be obtained as:

$${\hat{\boldsymbol{\upalpha }}}_{\text{RRcs}} = {\mathbf{U}}\hat{\mathbf{b}}_{\text{RRcs}} ,$$

where ${\mathbf{U}}$ is an $N \times N$ diagonal matrix, with diagonal values of $\frac{1}{{\sqrt {2p_{j} \left( {1 - p_{j} } \right)} }}$.

In the above paragraphs, we describe SNP-BLUP models but for convenience, we applied GREML models with back-solving to obtain the results (see “Appendix”). These two SNP-BLUP models are equivalent to GREML models with a centered GRM following VanRaden’s [10] method (1), and a centered and scaled GRM following VanRaden’s [10] method (2). In the case of GREML, the DGV must be back-solved to obtain the estimated regression coefficients for the SNP and, for the scaled GRM, the transformation to ASE is needed (see “Appendix”).

### Relationship between ASE from unscaled and scaled allele counts

First, we empirically evaluated the relationship between ASE from RRc and RRcs. For variants with the same MAF, the correlation, mean ratio and the regression coefficient were calculated between the ASE obtained with RRc and RRcs. The mean ratio was calculated by dividing the ASE estimated with RRcs by the ASE estimated with RRc for each SNP and averaged over all SNPs with the same MAF. Regression coefficients were obtained per set of SNPs with the same MAF by regressing the ASE from RRcs on the ASE from RRc. The number of SNPs per MAF ranged from 14 to 1796 with an average of 94 SNPs.

Second, we theoretically evaluated the expected relationship between ASE from RRc and RRcs. We considered expressions for estimating the ASE directly for a single locus, and ignored possible covariances between estimated ASE of different loci, which may for instance arise due to LD between the loci. For RRc, the equivalent ridge regression BLUP model can be specified as in e.g., [15, 20]:

$$\left( {{\mathbf{Z}}^{\prime }{\mathbf{D}}^{ - 1} {\mathbf{Z}} + \lambda_{RRc} } \right){\hat{\mathbf{b}}}_{{{\mathbf{RRc}}}} = {\mathbf{Z}}^{\prime }{\mathbf{D}}^{ - 1} {\mathbf{y}},$$

where $\lambda_{RRc} = \frac{{\sigma_{e}^{2} }}{{\sigma_{{\alpha_{RRc} }}^{2} }}$, with $\sigma_{{\alpha_{RRc} }}^{2}$ as SNP variance, i.e., $\sigma_{{\alpha_{RRc} }}^{2} = \frac{{\sigma_{a}^{2} }}{{ \sum \nolimits_{j} 2p_{j} \left( {1 - p_{j} } \right)}}$, with $\sigma_{a}^{2}$ as total additive genetic variance. Ignoring off-diagonal elements in ${\mathbf{Z^{\prime}D}}^{ - 1} {\mathbf{Z}}$, and using vector ${\mathbf{z}}_{.j}$ that is column $j$ in matrix ${\mathbf{Z}}$, we get:

$${\hat{\text{b}}}_{{{\text{RRc}},j}} = {\hat{{\upalpha }}}_{{{\text{RRc}},j}} = \frac{{{\mathbf{z}}_{.j}^{'} {\mathbf{D}}^{ - 1} {\mathbf{y}}}}{{{\mathbf{z}}_{.j}^{'} {\mathbf{D}}^{ - 1} {\mathbf{z}}_{.j} + \lambda_{RRc} }}.$$

Similarly, we can derive for RRcs:

$${\hat{\text{b}}}_{{{\text{RRcs}},j}} = {\hat{\upalpha }}_{RRcs,j} \sqrt {2p_{j} \left( {1 - p_{j} } \right)} = \frac{{{\mathbf{w}}_{.j}^{'} {\mathbf{D}}^{ - 1} {\mathbf{y}}}}{{{\mathbf{w}}_{.j}^{'} {\mathbf{D}}^{ - 1} {\mathbf{w}}_{.j} + \lambda_{RRcs} }},$$

where vector ${\mathbf{w}}_{.j}$ is column $j$ in matrix ${\mathbf{W}}$ and $\lambda_{RRcs} = \frac{{\sigma_{e}^{2} }}{{\sigma_{{\alpha_{RRcs} }}^{2} }}$, with $\sigma_{{\alpha_{RRcs} }}^{2}$ as SNP variance, i.e., $\sigma_{{\alpha_{RRcs} }}^{2} = \frac{{\sigma_{a}^{2} }}{N}$, and:

$${\hat{{\upalpha }}}_{{{\text{RRcs}},j}} = \frac{{{\hat{\text{b}}}_{{{\text{RRcs}},j}} }}{{\sqrt {2p_{j} \left( {1 - p_{j} } \right)} }} = \frac{{{\mathbf{w}}_{.j}^{'} {\mathbf{D}}^{ - 1} {\mathbf{y}}}}{{\left( {{\mathbf{w}}_{.j}^{'} {\mathbf{D}}^{ - 1} {\mathbf{w}}_{.j} + \lambda_{RRcs} } \right)\sqrt {2p_{j} \left( {1 - p_{j} } \right)} }} = \frac{{{\mathbf{z}}_{.j}^{'} {\mathbf{D}}^{ - 1} {\mathbf{y}}}}{{{\mathbf{z}}_{.j}^{'} {\mathbf{D}}^{ - 1} {\mathbf{z}}_{.j} + \lambda_{RRcs} \left[ {2p_{j} \left( {1 - p_{j} } \right)} \right]}}.$$

Thus, the ratio between both ASE, is equal to:

$$\begin{aligned} {\hat{{\upalpha }}}_{{{\text{RRcs}},j}} & = \frac{{\left( {{\mathbf{z}}_{.j}^{'} {\mathbf{D}}^{ - 1} {\mathbf{z}}_{.j} + \lambda_{RRc} } \right)}}{{\left( {{\mathbf{z}}_{.j}^{'} {\mathbf{D}}^{ - 1} {\mathbf{z}}_{.j} + \lambda_{RRcs} \left[ {2p_{j} \left( {1 - p_{j} } \right)} \right]} \right)}}{\hat{{\upalpha }}}_{RRc,j} , \\ {\hat{{\upalpha }}}_{{{\text{RRcs}},j}} & = \frac{{\left( {\frac{{\sigma_{a}^{2} }}{{\sigma_{e}^{2} }}{\mathbf{z}}_{.j}^{'} {\mathbf{D}}^{ - 1} {\mathbf{z}}_{.j} + \sum \nolimits_{k = 1}^{N} 2p_{k} \left( {1 - p_{k} } \right)} \right)}}{{\left( {\frac{{\sigma_{a}^{2} }}{{\sigma_{e}^{2} }}{\mathbf{z}}_{.j}^{'} {\mathbf{D}}^{ - 1} {\mathbf{z}}_{.j} + 2p_{j} \left( {1 - p_{j} } \right)N} \right)}}{\hat{{\upalpha }}}_{{{\text{RRc}},j}} . \\ \end{aligned}$$

Assuming that there is no relationship between the genotypes of the individuals and the information content of their phenotypes, e.g., the number of daughters in our study as represented in ${\mathbf{D}}^{ - 1}$, and that the genotypes are in Hardy–Weinberg equilibrium, ${\mathbf{z}}_{{.\varvec{j}}}^{\varvec{'}} {\mathbf{D}}^{ - 1} {\mathbf{z}}_{.j} \approx \mathop \sum _{i} \# dtrs_{i} 2p_{j} \left( {1 - p_{j} } \right)$, where $\# dtrs_{i}$ is the number of daughters in our case. The latter term could be replaced in other cases for instance by the (effective) number of own records, or if the individuals involved have only one own observation, then this term can simply be replaced by $n$ (number of individuals). In the latter situation, we get:

$$\begin{aligned} {\hat{{\upalpha }}}_{{{\text{RRcs}},j}} & = \frac{{\left( {\frac{{h^{2} }}{{1 - h^{2} }}n2p_{j} \left( {1 - p_{j} } \right) + \mathop \sum \nolimits_{k = 1}^{N} 2p_{k} \left( {1 - p_{k} } \right)} \right)}}{{\left( {\frac{{h^{2} }}{{1 - h^{2} }}n2p_{j} \left( {1 - p_{j} } \right) + 2p_{j} \left( {1 - p_{j} } \right)N} \right)}}{\hat{{\upalpha }}}_{{{\text{RRc}},j}} \\ & = \frac{{\left( {\frac{{h^{2} }}{{1 - h^{2} }}n + \frac{{\mathop \sum \nolimits_{k = 1}^{N} 2p_{k} \left( {1 - p_{k} } \right)}}{{2p_{j} \left( {1 - p_{j} } \right)}}} \right)}}{{\left( {\frac{{h^{2} }}{{1 - h^{2} }}n + N} \right)}}{\hat{{\upalpha }}}_{RRc,j} . \\ \end{aligned}$$

The value of the term $\mathop \sum _{j} 2p_{j} \left( {1 - p_{j} } \right)$ can be obtained as:

$$\mathop \sum _{j} 2p_{j} \left( {1 - p_{j} } \right) = N \cdot {\text{E}}\left[ {2p_{.} \left( {1 - p_{.} } \right)} \right]$$

and

$${\text{E}}\left[ {2p_{.} \left( {1 - p_{.} } \right)} \right] = \sum _{{p = \frac{1}{2n}}}^{{\frac{2n - 1}{2n}}} 2p\left( {1 - p} \right)\phi \left( p \right),$$

where $\phi \left( p \right)$ is the probability density function of the distribution of the allele frequencies, which is required. Here, we consider two distributions, i.e., the uniform distribution, which generally applies for commonly used 50 K SNP chips [21, 22] and the U-shaped distribution, which applies to whole-genome sequence data [21, 23, 24].

For the uniform distribution, incrementing $p$ by steps of $\frac{1}{2n}$, we obtain:

$$\begin{aligned} {\text{E}}\left[ {2p_{.} \left( {1 - p_{.} } \right)} \right] & = \mathop \sum \limits_{{p = \frac{1}{2n}}}^{{\frac{2n - 1}{2n}}} 2p\left( {1 - p} \right)\phi \left( p \right) \\ & \approx \mathop \int \limits_{0}^{1} 2p\left( {1 - p} \right)\frac{1}{1 - 0}dp = F\left( 1 \right) - F\left( 0 \right) = \frac{1}{3}, \\ \end{aligned}$$

since the derivative is obtained as $F\left( {2p\left( {1 - p} \right)} \right) = p^{2} - \frac{2}{3}p^{3}$.

Thus, when the allele frequencies are uniformly distributed:

$${\hat{{\upalpha }}}_{{{\text{RRcs}},j}} = \frac{{\left( {\frac{{h^{2} }}{{1 - h^{2} }}n + \frac{{\frac{1}{3}N}}{{2p_{j} \left( {1 - p_{j} } \right)}}} \right)}}{{\left( {\frac{{h^{2} }}{{1 - h^{2} }}n + N} \right)}}{\hat{{\upalpha }}}_{{{\text{RRc}},j}} .$$

For the U-shaped distribution, the probability density function, is $\phi \left( p \right) \approx Cp^{{4N_{e} v - 1}} \left( {1 - p} \right)^{{4N_{e} u - 1}} e^{{4N_{e} sp\left( {1 - p} \right)}}$ [25], where $v$ and $u$ are assumed to be equal and represent forward and backward mutation rates (here assumed to be $1 \times 10^{ - 8}$), $N_{e}$ is the effective population size (here assumed to be 65), $s$ is the selection coefficient and $C$ is a constant that scales the sum of all probabilities to 1. Assuming $s = 0$ for simplification, the term $e^{{4N_{e} sp\left( {1 - p} \right)}}$ drops from the equation, such that $\phi \left( p \right) \approx Cp^{{4N_{e} v - 1}} \left( {1 - p} \right)^{{4N_{e} v - 1}}$. In this case,

$$C = \left( {\mathop \sum \limits_{{p = \frac{1}{2n}}}^{{\frac{2n - 1}{2n}}} p^{{4N_{e} v - 1}} \left( {1 - p} \right)^{{4N_{e} v - 1}} } \right)^{ - 1} .$$

Thus, we get:

$$\begin{aligned} {\text{E}}\left[ {2p_{.} \left( {1 - p_{.} } \right)} \right] & = \mathop \sum \limits_{{p = \frac{1}{2n}}}^{{\frac{2n - 1}{2n}}} 2p\left( {1 - p} \right)Cp^{{4N_{e} v - 1}} \left( {1 - p} \right)^{{4N_{e} v - 1}} \\ & = 2C\mathop \sum \limits_{{p = \frac{1}{2n}}}^{{\frac{2n - 1}{2n}}} p^{{4N_{e} v}} \left( {1 - p} \right)^{{4N_{e} v}} . \\ \end{aligned}$$

Substituting this in the earlier formula, we get for the U-shaped distribution:

$${\hat{{\upalpha }}}_{{{\text{RRcs}},j}} = \frac{{\left( {\frac{{h^{2} }}{{1 - h^{2} }}n + \frac{{2NC\mathop \sum \nolimits_{{p = \frac{1}{2n}}}^{{\frac{2n - 1}{2n}}} p^{{4N_{e} v}} \left( {1 - p} \right)^{{4N_{e} v}} }}{{2p_{j} \left( {1 - p_{j} } \right)}}} \right)}}{{\left( {\frac{{h^{2} }}{{1 - h^{2} }}n + N} \right)}}{\hat{{\upalpha }}}_{{{\text{RRc}},j}} .$$

This expression is rather tedious. Here, we assumed that $v = 1 \times 10^{ - 8}$, and the effective population size $N_{e} = 65$ [26] resulting in $4N_{e} v = 2.6 \times 10^{ - 6} \approx 0$. Hence, in our case, and in other datasets where $N_{e}$ is small such that $4N_{e} v \approx 0$, and considering that $n$ is not extremely large, $C$ can be approximated as:

$$\begin{aligned} C^{*} & = \left( {\mathop \sum \limits_{{p = \frac{1}{2n}}}^{{\frac{2n - 1}{2n}}} p^{{4N_{e} v - 1}} \left( {1 - p} \right)^{{4N_{e} v - 1}} } \right)^{ - 1} \\ & \approx \frac{1}{4n}\left( {\ln \left( {2n - 1} \right) + \frac{1}{4n - 2} + {{\upgamma }}} \right)^{ - 1} , \\ \end{aligned}$$

where $\gamma$ is the Euler–Mascheroni constant [27] (see “Appendix” for a derivation).

Thus, for situations where $4N_{e} v \approx 0$, and $n$ is not extremely large, we get:

$$\begin{aligned} {\text{E}}\left[ {2p_{.} \left( {1 - p_{.} } \right)} \right] & = \mathop \sum \nolimits_{{p = \frac{1}{2n}}}^{{\frac{2n - 1}{2n}}} 2p\left( {1 - p} \right)C^{*} p^{{4N_{e} v - 1}} \left( {1 - p} \right)^{{4N_{e} v - 1}} \\ & = 2C^{*} \mathop \sum \nolimits_{{p = \frac{1}{2n}}}^{{\frac{2n - 1}{2n}}} p^{{4N_{e} v}} \left( {1 - p} \right)^{{4N_{e} v}} . \\ \end{aligned}$$

Given the value of $4N_{e} v$ used here, $\sum\nolimits_{{p = \frac{1}{2n}}}^{{\frac{2n - 1}{2n}}} p^{{4N_{e} v}} \left( {1 - p} \right)^{{4N_{e} v}} \approx 2n - 1$, such that:

$${\text{E}}\left[ {2p_{.} \left( {1 - p_{.} } \right)} \right] \approx 2C^{*} \left( {2n - 1} \right).$$

Substituting this in the earlier formula, we get for the U-shaped distribution:

$${\hat{{\upalpha }}}_{{{\text{RRcs}},j}} = \frac{{\left( {\frac{{h^{2} }}{{1 - h^{2} }}n + \frac{{NC^{*} \left( {2n - 1} \right)}}{{p_{j} \left( {1 - p_{j} } \right)}}} \right)}}{{\left( {\frac{{h^{2} }}{{1 - h^{2} }}n + N} \right)}}{\hat{{\upalpha }}}_{{{\text{RRc}},j}} .$$

The above formulae show that under the assumption that the ASE are not affected by LD with other SNPs, the ratio between ${\text{E}}\left( {{\hat{{\upalpha }}}_{RRc,j} } \right)$ and ${\text{E}}({\hat{{\upalpha }}}_{RRcs,j} )$ is the result of shrinkage, i.e., when the amount of information used becomes (very) large (i.e., many individuals), ${\hat{{\upalpha }}}_{RRc,j}$ and ${\hat{{\upalpha }}}_{RRcs,j}$ will be the same.

### Optimal scaling parameter

To determine the optimal scaling parameter, both the fit of the model to the data and the predictive performance of the model were evaluated with different scaling parameters. Adopting the notation by Speed et al. [28], allele counts from matrix ${\mathbf{X}}$ were recoded resulting in matrix ${\mathbf{V}}$ with elements computed as $v_{ij} = \left( {x_{ij} - 2p_{j} } \right) \times \left( {2p_{j} \left( {1 - p_{j} } \right)} \right)^{\gamma /2}$. Hence the SNP-BLUP model became ${\mathbf{y}} = \mathbf{1}\mu + {\mathbf{Vb}}_{\gamma } + {\mathbf{e}}$. Different scaling parameters were tested by varying $\gamma$ from − 1 [i.e., scaling by the standard deviation (RRcs)] to 0 [i.e., no scaling (RRc)] in steps of 0.1 for elements of the matrix ${\mathbf{V}}$.

The model fit was evaluated by comparing the log-likelihoods from the SNP-BLUP models using the complete stature dataset.

The mean squared error of prediction (MSEP) was evaluated by running the SNP-BLUP model with the different scaling parameters ($\gamma$) using the stature dataset, which was split in a training (3414 older bulls) and validation set (2140 young bulls). The MSEP for the validation animals was calculated as $\frac{{\mathop \sum \nolimits_{i}^{{}} \left( {wt_{i} \times \left( {DGV_{i} + \mu - DYD_{i} } \right)^{2} } \right)}}{{\mathop \sum \nolimits_{i}^{{}} wt_{i} }}$, with $wt_{i}$ being the number of daughters of bull $i$ included in the $DYD_{i}$, which was used as the actual phenotype for stature for validation bull *i*, $DGV_{i} + \mu$ being the DGV of bull $i$ plus the general mean which together result in the predicted phenotype (i.e., $\hat{y}$) for the validation bull $i$. The model with the lowest MSEP was considered to be the most appropriate for genomic prediction, and thus also for estimating ASE.

## Results

Using a reference dataset of 5554 bulls with HD genotypes (627,440 SNPs coded as 0, 1, 2) and stature phenotypes, we compared the ASE from a SNP-BLUP model using two commonly used genotype coding methods. The first method proposed (RRc) centers the 0, 1, 2 coded genotypes; the second method proposed (RRcs) centers and scales the 0, 1, 2 coded genotypes.

### Comparison of ASE from unscaled and scaled allele counts

**Fig. 2:**
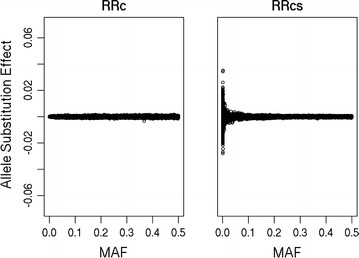
Allele substitution effects plotted against minor allele frequency (MAF)

**Fig. 3:**
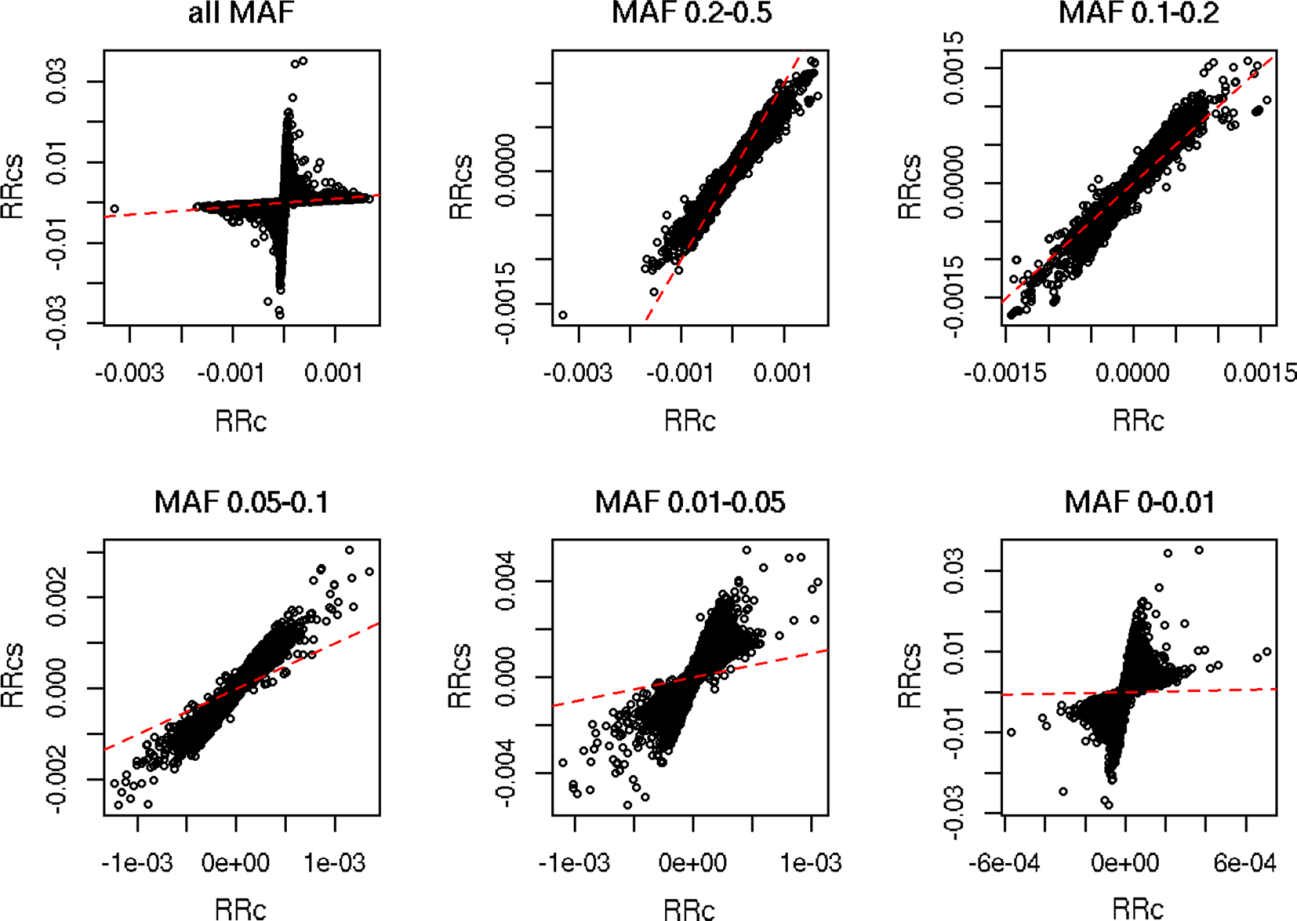
Allele substitution effects based on unscaled (RRc) and scaled (RRcs) allele counts categorized in minor allele frequency (MAF) classes

**Fig. 4:**
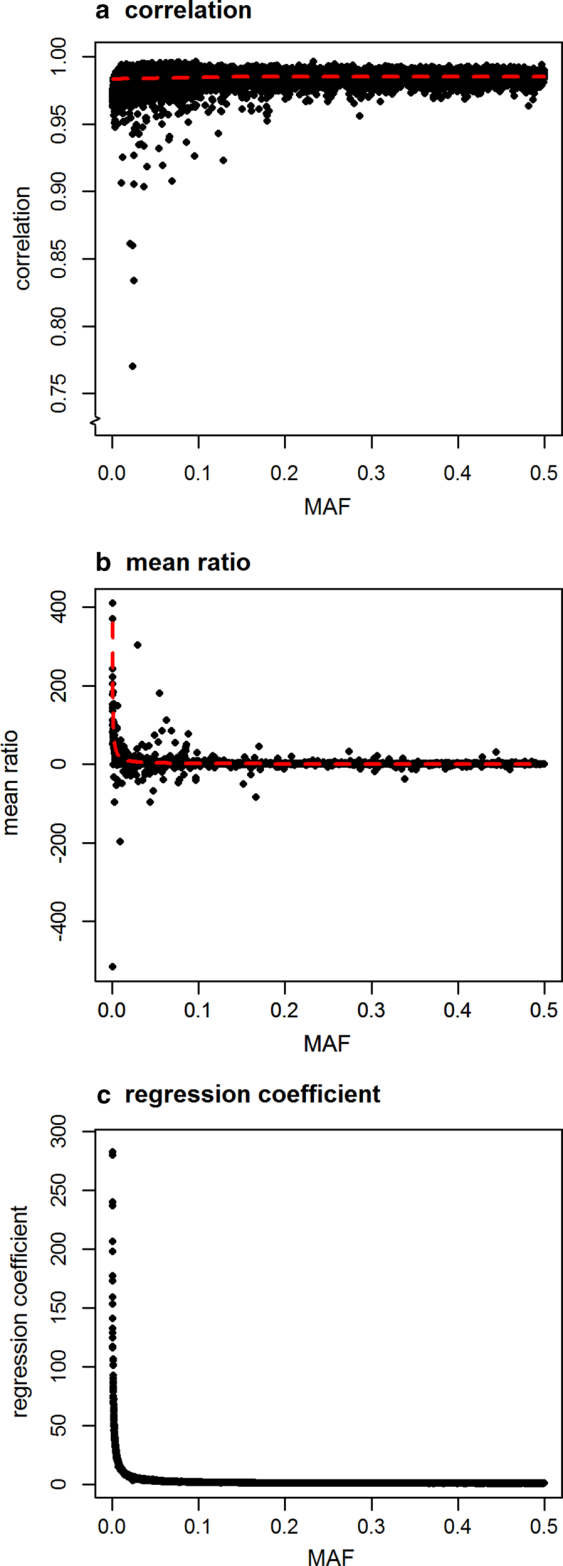
Correlation (**a**), mean ratio (**b**), and regression coefficient (**c**) between allele substitution effects based on unscaled (RRc) and scaled (RRcs) allele counts per minor allele frequency (MAF). For all SNPs with the same MAF the correlation between allele substitution effects (ASE) from RRc and RRcs are plotted in **a**. The red dashed line in **a** is a smoothed LOESS. The mean of the ratios between ASE from RRc and RRcs (ASE_RRcs_/ASE_RRc_) for SNPs with the same MAF are plotted in **b**. The red dashed line in **b** indicates the ratio between ASE based on RRc and RRcs using the derived equation for uniformly distributed MAF, given the MAF of the SNPs in the real data. For all SNPs with the same MAF, the regression coefficient for the regression of ASE from RRcs on ASE from RRc are plotted in **c**

The correlation between DGV using RRc and RRcs was equal to 0.9988 (regression coefficient (regression of RRcs on RRc) = 1.0011; $\sigma_{DGV}^{2} :RRc = 1.64, RRcs = 1.68$), whereas the correlation between ASE using RRc and RRcs was equal to 0.27. The main difference in ASE between the two methods was found for SNPs with a MAF lower than 0.01, as shown in Fig. 2. Figure 3 shows that although the overall correlation is low, there is a relationship between the ASE from RRc and RRcs. Please note the differences between the x-axis and y-axis in the different plots. As the MAF increases, the ASE from RRc and RRcs become more similar, and Fig. 3 shows that ASE from SNPs with a higher MAF are located closer to the diagonal. With lower MAF, the ASE from RRcs become larger than ASE from RRc, and Fig. 3 shows that ASE from SNPs with a lower MAF have much steeper regression coefficients. Although ASE seem to correlate poorly between RRc and RRcs, the correlation between ASE of variants with the exact same MAF was high, i.e., ranging from 0.770 to 0.996, with an average correlation of 0.98 (Fig. 4a). However, on the one hand, both the mean ratio (ASE from RRcs divided by ASE from RRc, averaged per MAF; Fig. 4b) and the regression coefficient (regression of ASE from RRcs on ASE from RRc; Fig. 4c) were much higher than 1 for low MAF variants. On the other hand, the mean ratio and regression coefficients were often lower than 1 for SNPs with a MAF higher than 0.25 (results not shown) because the total variance explained by all SNPs together remains the same. The differences in ASE between RRc and RRcs, and hence the observed mean ratios and regression coefficients, are due to the difference in shrinkage towards the mean. For RRc, the ASE of variants with a low MAF are heavily shrunk back towards the mean; this shrinkage is much less strong in RRcs due to the scaling of the allele counts, which considers the allele frequencies.

**Fig. 5:**
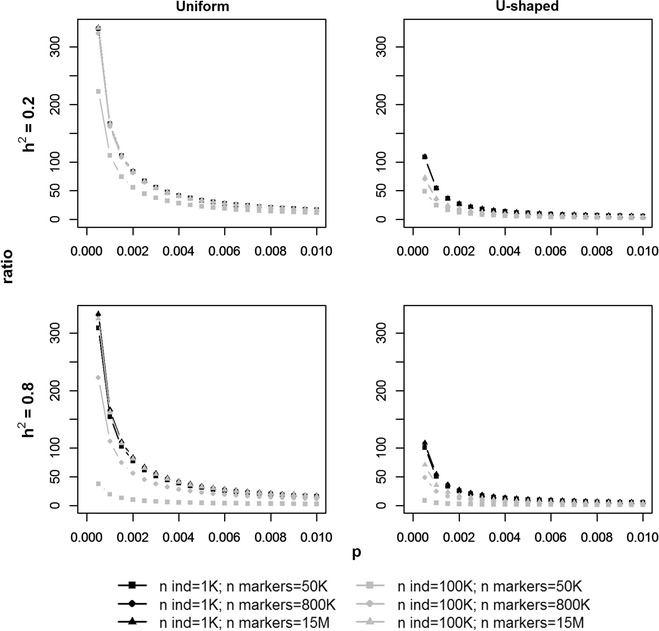
Theoretical ratio (ASE_RRcs_/ASE_RRc_) between allele substitution effects (ASE) based on unscaled (RRc) and scaled (RRcs) allele counts. Ratio for different allele frequencies (*p*) based on derivations for a uniform (left) and U-shaped (right) distribution of allele frequency for marker sets. Scenarios based on the number of individuals, i.e., 1000 (1 K; black) and 100,000 (100 K; gray); and number of SNPs, i.e., 50,000 (50 K; square), 800,000 (800 K; dot), 15,000,000 (15 M; triangle). Here, only allele frequencies lower than 0.01 are shown for heritabilities (h^2^) of 0.2 (first row) and 0.8 (second row)

Figure 4b shows the ratio between ASE based on RRc and RRcs in the real data for stature. The red dashed line in Fig. 4b indicates the ratio based on the derived equation for the uniform distribution of allele frequencies using the heritability, number of individuals and SNP as well as the frequencies of the real data, and this shows that the equation accurately fits the general trend of those mean ratios estimated from the real data. The regression coefficients in Fig. 4c also fit well with the ratios from the equation for the uniform distribution of allele frequencies, although the maximum regression coefficient (282) was lower than the maximum from the equation for the uniform distribution (360). Figure 5 shows the ratio as derived for different simulated scenarios for both the uniform and U-shaped distributions. Allele frequencies lower than 0.01 have high ratios for both allele frequency distributions (Fig. 5). Scenarios with a large number of individuals (100 K), a small number of SNPs (50 K) and a high heritability (0.8) have ratios closer to 1 at the extremely low MAF compared with the other scenarios (Fig. 5). The ratio of estimated ASE for the low MAF variants from the U-shaped distribution was substantially lower than that for the uniform distribution of allele frequencies.

For the uniform distribution, SNPs with an intermediate MAF of ~ 0.2 showed ratios around 1, and SNPs with a MAF of 0.5 had ratios below 1 with a minimum of 0.67 (h^2^ = 0.2, 1000 individuals, and 15 × 10^6^ SNPs), and a maximum of 0.96 (h^2^ = 0.8, 100,000 individuals, and 50 K SNPs). For the U-shaped distribution, ratios were around 1 for SNPs with a MAF of 0.065 for scenarios with 1000 individuals, and for SNPs with a MAF of 0.04 for scenarios with 100,000 individuals. At a MAF of 0.5, the U-shaped distribution reached ratios between 0.24 and 0.30 for scenarios with 1000 individuals, and between 0.16 (h^2^ = 0.2, 15 × 10^6^ SNPs) and 0.91 (h^2^ = 0.8, 50 K SNPs) for scenarios with 100,000 individuals.

### Optimal scaling parameter

We have shown, empirically and theoretically, that the scaling of allele counts influences the ASE of variants with a low MAF. Here, we attempted to determine which scaling parameter gave the best fit of the model to the data and which had the best predictive performance.

**Fig. 6:**
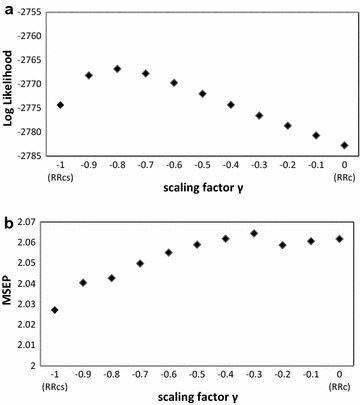
Log-likelihood (**a**) and mean squared error of prediction (MSEP) (**b**) from SNP-BLUP varying scaling from − 1 (RRcs) to 0 (RRc) in steps of 0.1

The optimal scaling parameter was determined by comparing the maximum likelihood for SNP-BLUP models for which the scaling parameter $\gamma$ varied from − 1 (i.e., RRcs) to 0 (i.e., RRc) in steps of 0.1. The model with a scaling parameter of $\gamma = - 0.8$ retained the highest log-likelihood (Fig. 6a), and was therefore the optimal scaling parameter for this dataset.

The optimal scaling parameter for predictive performance was determined by comparing the MSEP of the SNP-BLUP models for which scaling parameters varied from − 1 (i.e., RRcs) to 0 (i.e., RRc) in steps of 0.1. The stature dataset was split into a training set of 3414 older bulls and a validation set of 2140 young bulls for which the MSEP was assessed. The model with a scaling parameter of $\gamma = - 1$ (i.e., RRcs) retained the lowest MSEP (Fig. 6b), and therefore had the best predictive performance for this dataset.

## Discussion

The ASE indicates the effect of the variant on the phenotype under investigation. When searching for variants that cause variation in the phenotype, most of those detected are either common variants (MAF > 5%) or variants that explain a large proportion of the variance in the trait, e.g., Mendelian traits [29, 30]. The most difficult causal variants to detect are those with a low MAF (0.5% < MAF < 5%) and low to moderate effect, as well as those that are rare (MAF < 0.5%) [29, 30]. If the power of the study design is sufficient and the ASE moderate, genomic prediction models using such low MAF variants might explain part of the so-called missing heritability [29]. With whole-genome sequence data, low MAF variants and even rare variants can be identified and used in genomic prediction. However, in this study, we demonstrated that different genotype coding methods result in different estimated ASE, especially for low MAF variants. Due to the increased interest in low MAF variants with the advent of using whole-genome sequence data in genomic prediction, it will become increasingly important to determine how genotypes are coded prior to estimating the ASE. Here, we showed that scaling of allele counts (0, 1, 2 genotypes) influences the estimation of ASE of low MAF variants due to more (unscaled) or less (scaled) shrinkage towards the mean of the ASE. In addition, our results show that centered and scaled allele counts (e.g., RRcs) put more weight on low MAF variants, which suggests that scaling is more preferable for long-term genomic selection than no scaling (e.g., RRc) [15, 16]. Although these results were expected based on the underlying model assumptions, our aim was to visualize the differences in ASE between scaling and no scaling, to provide a theoretical framework, and create awareness of the implications for future studies, in animal and plant breeding, and even in genomic prediction of disease risk in humans. Our empirical results closely resemble the expectation based on the presented theoretical framework. In practice, accurately estimating the ASE of low MAF variants remains difficult and requires powerful study designs.

### Scaling

Although in this study we presented SNP-BLUP (or ridge regression BLUP) models, equivalent models that do not explicitly estimate ASE, i.e., GREML or GBLUP, will similarly result in different estimates of the ASE when the allele counts used in the construction of a GRM are scaled or not. The same applies to other regression-based models that include parameters that accommodate for differential shrinkage across loci, e.g., Bayesian variable selection models such as BayesC and BayesR. For those models, the difference in ASE when scaling is used or not may be smaller than for GBLUP and SNP-BLUP, because these differential shrinkage models have additional parameters to modify shrinkage per locus.

Optimal scaling depends on the true relationship between the ASE size and MAF in the data analyzed [31]. Causal alleles with a larger effect tend to have a lower MAF [32], hence for models with scaled allele counts that give low MAF variants a larger ASE (i.e., RRcs) may represent more closely the truth. Speed et al. [31] showed that scaling by the square root of the variance (i.e., RRcs; $\gamma = - 1$) gave stable results in estimating the heritability (based on SNPs), regardless of the simulated genetic structure of the trait. In our study, we showed that a scaling parameter of $\gamma = - 0.8$, gave the best fit of the model to the data, whereas a scaling parameter of $\gamma = - 1$ (i.e., RRcs) gave the best predictive performance in terms of MSEP (Fig. 6), which suggests that RRcs gave better ASE estimates than RRc for our dataset. In a recent paper, Speed et al. [28] re-evaluated the scaling parameters for scenarios that include low MAF variants. They recommended a scaling parameter of $\gamma = - 0.25$ when low MAF variants are included, while the estimated heritabilities (based on SNPs) were hardly influenced by the scaling parameter when analyzing only common SNPs. In their analysis, they used a model with two GRM, one for SNPs with a MAF lower than 0.1 and one for SNPs with a MAF higher than 0.1, versus a model with one GRM containing all SNPs. The scaling parameter that fitted equally well, based on the REML likelihood in both models, was recommended. In our study, the optimal scaling parameter for this dataset was tested with the aim of maximizing model fit and minimizing the MSEP for the validation data. In our case, differences in log-likelihood and MSEP among the scaling parameters applied were rather small. The optimal scaling parameter may also depend on the characteristics of the data, such as the MAF spectrum. Here, the genotypes were from the BovineHD SNP chip with 630,000 SNPs, which contained a fair number of SNPs with a MAF lower than 1% (5% of the SNPs) and even rare SNPs with a MAF lower than 0.5% (3.7% of the SNPs) (Fig. 1); future studies focusing on whole-genome sequence variants might contain even more low MAF variants, which might result in a different optimal scaling parameter.

### Allele frequency distribution

The derived equations for the ratios of ASE from a uniform and U-shaped allele frequency distribution show the implications of (more/less) shrinkage towards the mean for different scenarios. The heritability, number of individuals, and the number of variants all have an impact on the ratio. For both allele frequency distributions, with a small number of individuals (i.e., 1000) there seems to be little impact of heritability and number of variants on the difference in shrinkage between scaling and no scaling. However, when the number of individuals is large (i.e., 100,000), it is clear that heritability and number of variants have an impact.

For variants with a low MAF, the ASE obtained with RRcs and RRc are more similar when the number of variants is small and especially when the number of individuals is large and the heritability is high. These results can be interpreted as follows. Differences in genotype coding effectively reflect a difference in prior belief of the impact of rare versus more common variants. RRcs assumes that rare variants are more important than common ones, and therefore, in many cases, the shrinkage of effects of low MAF variants is much lower for RRcs than for RRc. The data can override this prior information, if the dataset is sufficiently large. All possible aspects of the dataset that increase power, i.e., more animals, higher heritability and fewer variants, all reduced the ratios between both ASE, showing that shrinkage due to the prior assumptions of the models was reduced. In other words, the prior assumptions made when choosing the genotype coding become less important when the power of the data increases. However, when increasing power by increasing the population size, even more rare variants will appear with an even lower MAF than what was considered as rare beforehand. This is because rare variants were defined in terms of MAF instead of number of alleles observed. For those ‘newly’ discovered rare variants, the ratio between RRcs and RRc will be high again and the power will be too low to estimate their effects very accurately, hence power should be analyzed to determine the MAF at which the ASE can be estimated accurately given the data.

Remarkably, in all scenarios, the U-shaped allele frequency distribution showed lower ratios for low MAF variants compared to the uniform allele frequency distribution. This is probably because a U-shaped distribution has more low MAF variants, and also since the total variance remains the same, there is less room for the model to allocate an extremely high ASE for all those low MAF alleles.

The distribution of the allele frequencies for the real data with HD genotypes was not uniform, but more like the U-shape distribution (Fig. 6). This more U-shaped distribution for the HD genotypes is not only true for this dataset. This is due to the chip design, which allowed for more low MAF variants to be included compared to the BovineSNP50 chip. However, the ratios in ASE between RRcs and RRc for real data on stature in cattle were better aligned with the derived ratios for a uniform distribution. The most logical explanation is that the U-shaped distribution that we assumed resembles that of whole-genome sequence data, where the number of low MAF variants is relatively much larger than for the high-density chip data that we used. Effectively, the allele frequency distribution in our chip data may be closer to the uniform distribution than the considered U-shaped distribution. Theoretically, it should be possible to use the formula that was used for the U-shaped distribution to represent any other possible allele frequency distribution, by tuning its parameters. This would provide a more general applicable theoretical framework to predict the impact of different genotype coding on the shrinkage of estimated effects, if those parameters can be computed or derived empirically.

To derive the ratio of ASE from RRcs and RRc for both allele frequency distributions, we made several assumptions. One of these assumptions was that there are no covariances between the estimated ASE of different loci, however, covariances may, for instance, arise due to LD between the loci. Especially for the U-shape distribution, which represents whole-genome sequence data, high LD between variants is expected. In that case, the assumption “ignoring off-diagonal elements in ${\mathbf{Z^{\prime}R}}^{ - 1} {\mathbf{Z}}$” (which is the same as ignoring LD between loci) is likely to be violated when allele coding is not independent of the MAF (e.g., minor allele counted). Nevertheless, the theoretical ratios based on the uniform distribution fitted nicely with the actual ratios from the real stature data example and did not seem to be hampered by the presence of LD (Fig. 4b).

## Conclusions

The results of our study show that DGV are not influenced by scaling of centered allele counts, while the estimates of ASE are. Large differences in ASE between scaled and unscaled allele counts were observed for variants with a low MAF, mainly due to less shrinkage towards the mean for scaled allele counts. We derived a theoretical framework that shows that the difference in ASE due to (more/less) shrinkage is heavily influenced by the power of the data. Increasing the power, by increasing the number of animals, increasing the heritability or decreasing the number of variants, resulted in smaller differences of the ASE between scaled and unscaled allele counts.

## Appendix

### GREML

In this paper, we describe SNP-BLUP models, however GREML/GBLUP models are equivalent and were actually applied to the data. This appendix contains the methods for genomic evaluation followed by the back-solving method to obtain allele substitution effects using GREML with a centered GRM according to Method (1) in VanRaden [1] (VR1; equivalent to RRc) and a centered and scaled GRM according to Method (2) in VanRaden [1] (VR2; equivalent to RRcs). We also provide the method to construct GRM with varying scaling factors for GREML (or GBLUP) models.

#### Genomic evaluation

To estimate DGV, GREML was performed using two GRM constructed in different ways to show the impact of scaling on ASE. The following mixed model equation was solved using GREML in ASReml software [2]:

$$y_{\varvec{i}} = \mu + u_{i} + e_{i} ,$$

where $y_{\varvec{i}}$ is the phenotypic record, here stature DYD, of individual $i$; $\mu$ is the mean; $u_{i}$ is the DGV of individual $i$, with $u\sim N\left( {0,{\mathbf{G}}\sigma_{a}^{2} } \right)$, where ${\mathbf{G}}$ is the genomic relationship matrix, and $\sigma_{a}^{2}$ the total additive genetic variance; and $e_{i}$ is the residual of individual $i$, with $e\sim N\left( {0,{\mathbf{D}}\sigma_{e}^{2} } \right)$, where ${\mathbf{D}}$ is a diagonal matrix with elements computed as $\frac{1}{{wt_{i} }}$, with $wt_{i}$ being the number of daughters of individual $i$ on which the DYD of $i$ was based as weight, and $\sigma_{e}^{2}$ is the residual variance.

The different GRM used were:

VR1 [Method (1) according to VanRaden [1]; equivalent to the SNP-BLUP model with centered allele counts (RRc)]:

$${\mathbf{G}}_{{{\text{VR}}1}} = \frac{{{\mathbf{ZZ^{\prime}}}}}{{2\mathop \sum \nolimits_{\varvec{j}} p_{j} \left( {1 - p_{j} } \right)}},$$

where ${\mathbf{Z}}$ contains the centered allele counts of the SNP, with elements computed as $x_{ij} - 2p_{j}$, where $x_{ij}$ is an element of the ${\mathbf{X}}$ matrix containing the SNP genotype for individual $i$ at locus $j$ coded as 0, 1, or 2; and $p_{j}$ is the frequency of the allele for which the homozygous genotype is coded as 2 at locus $j$. Note that $2p_{j}$ is the mean allele count of the SNP for the centering.

VR2 [Method (2) according to VanRaden [1]; equivalent to the SNP-BLUP model with centered and scaled allele counts (RRcs)]:

$${\mathbf{G}}_{{{\text{VR}}2}} = \frac{{{\mathbf{WW^{\prime}}}}}{N},$$

where $N$ is the number of SNPs; and ${\mathbf{W}}$ contains the centered and scaled allele counts of the SNPs for all individuals at all loci, with elements computed as $w_{ij} = \frac{{\left( {x_{ij} - 2p_{j} } \right)}}{{\sqrt {2p_{j} \left( {1 - p_{j} } \right)} }}$. Note that $\sqrt {2p_{j} \left( {1 - p_{j} } \right)}$ is the standard deviation used for the scaling.

#### Back-solving

Considering VR1, the ASE (${\hat{\boldsymbol{\upalpha }}}_{{{\text{VR}}1}}$) were back-solved as coefficients of the regression of the DGV (${\hat{\mathbf{u}}}_{{{\text{VR}}1}}$) on the allele counts. Considering that ${\hat{\mathbf{u}}}_{{{\text{VR}}1}} = {\mathbf{Z}\hat{\boldsymbol{\upalpha }}}_{{{\text{VR}}1}}$ yields the following expression [3]:

$${\hat{\boldsymbol{\upalpha }}}_{{{\text{VR}}1}} = {\mathbf{Z^{\prime}}}\left( {{\mathbf{ZZ^{\prime}}}} \right)^{ - 1} {\hat{\mathbf{u}}} = S^{ - 1} {\mathbf{Z^{\prime}G}}_{{{\text{VR}}1}}^{ - 1} {\hat{\mathbf{u}}},$$

where $$S = \mathop \sum_{j} 2p_{j} \left( {1 - p_{j} } \right)$$.

For VR2, the following applies: ${\hat{\mathbf{u}}}_{{{\text{VR}}2}} = {\mathbf{W}\hat{\mathbf{b}}}_{{{\text{VR}}2}}$, where ${\hat{\mathbf{b}}}_{{{\text{VR}}2}}$ are regression coefficients, that can be obtained with:

$${\hat{\mathbf{b}}}_{{{\text{VR}}2}} = {\mathbf{W^{\prime}}}\left( {{\mathbf{WW^{\prime}}}} \right)^{ - 1} {\hat{\mathbf{u}}} = N^{ - 1} {\mathbf{W}}^{'} {\mathbf{G}}_{{{\text{VR}}2}}^{ - 1} {\hat{\mathbf{u}}}.$$

Note that the ${\hat{\mathbf{b}}}_{{{\text{VR}}2}}$ values are not allele substitution effects, i.e., they are not equal to half the difference of the value between the two homozygotes [4], because ${\mathbf{W^{\prime}}}$ contains scaled allele counts. The allele substitution effects for VR2 (${\hat{\boldsymbol{\upalpha }}}_{{{\text{VR}}2}} )$ can be obtained as:

$${\hat{\boldsymbol{\upalpha }}}_{{{\text{VR}}2}} = {\mathbf{U}\hat{\mathbf{b}}}_{{{\text{VR}}2}} ,$$

where ${\mathbf{U}}$ is an $N \times N$ diagonal matrix, with diagonal values of $\frac{1}{{\sqrt {2p_{j} \left( {1 - p_{j} } \right)} }}$. Thus, following the above definition, DGV based on VR2 can be computed as ${\hat{\mathbf{u}}}_{{{\text{VR}}2}} = {\mathbf{Z}\hat{\boldsymbol{\upalpha }}}_{{{\text{VR}}2}}$.

The back-solving procedure was verified, within each method, by recalculating the DGV as ${\hat{\mathbf{u}}} = {\mathbf{Z}\hat{\boldsymbol{\upalpha }}}$, i.e., the back-solved ASE were multiplied with the 0, 1, 2 coded genotypes (i.e., allele counts) and the appropriate general mean was added, and then compared to the original DGV. This gave for both methods correlations and regression coefficients of exactly 1, which demonstrates that the back-solving procedures were correct.

#### Optimal scaling parameter

Adopting the notation of Speed et al. [5], GRM were constructed using matrix ${\mathbf{V}}$ with elements computed as $v_{ij} = \left( {x_{ij} - 2p_{j} } \right) \times \left( {2p_{j} \left( {1 - p_{j} } \right)} \right)^{\gamma /2}$, and ${\mathbf{G}} = {\mathbf{VV^{\prime}}} \times (\mathop \sum _{j} (2p_{j} \left( {1 - p_{j} } \right)))^{{\left( { - 1 - \gamma } \right)}}$ by varying $\gamma$ from − 1 (i.e., VR2) to 0 (i.e., VR1) in steps of 0.1. The factor used to compute ${\mathbf{G}}$, i.e., $(\mathop \sum _{j} (2p_{j} \left( {1 - p_{j} } \right)))^{{\left( { - 1 - \gamma } \right)}}$, is derived as follows. With proper scaling of ${\mathbf{G}}$, and assuming that allele frequencies used are computed from the current population, the average inbreeding is expected to be zero, hence ${\text{E}}\left( {trace\left( {\mathbf{G}} \right)} \right) = n$, where $n$ is the number of individuals. Consider that the variance of column $j$ in ${\mathbf{V}}$ is $var\left( {{\mathbf{V}}_{{.{\mathbf{j}}}} } \right) = \varvec{v}ar\left( {{\mathbf{X}}_{{.{\mathbf{j}}}} } \right) \times \left( {2p_{j} \left( {1 - p_{j} } \right)} \right)^{\gamma } = 2p_{j} \left( {1 - p_{j} } \right) \times \left( {2p_{j} \left( {1 - p_{j} } \right)} \right)^{\gamma } = \left( {2p_{j} \left( {1 - p_{j} } \right)} \right)^{{\left( {1 + \gamma } \right)}}$. Consequently, ${\text{E}}\left( {trace\left( {{\mathbf{VV}}'} \right)} \right) = {\text{E}}\left( {\mathop \sum _{i} \mathop \sum _{j} v_{ij}^{2} } \right) = {\text{E}}\left( {n\mathop \sum _{j} var\left( {{\mathbf{V}}_{{.{\mathbf{j}}}} } \right)} \right) = n(\mathop \sum _{j} (2p_{j} \left( {1 - p_{j} } \right)))^{{\left( {1 + \gamma } \right)}}$. To get ${\text{E}}\left( {trace\left( {\mathbf{G}} \right)} \right) = n$, then requires to multiply ${\mathbf{VV}}'$ by $(\mathop \sum _{j} (2p_{j} \left( {1 - p_{j} } \right)))^{{\left( { - 1 - \gamma } \right)}}$. For all scaling parameters, the diagonal elements of the GRM were on average 1, resulting in an average inbreeding coefficient of 0.

### Derivation of $C$ for the U-shape distribution when $4N_{e} v \approx 0$

For the U-shaped distribution, the probability density function, is $\phi \left( p \right) \approx Cp^{{4N_{e} v - 1}} \left( {1 - p} \right)^{{4N_{e} u - 1}} e^{{4N_{e} sp\left( {1 - p} \right)}}$ [6], where $v$ and $u$ are assumed to be equal and represent forward and backward mutation rates, $N_{e}$ is the effective population size, $s$ is the selection coefficient and $C$ is a constant that scales the sum of all probabilities to 1. Assuming $s = 0$ for simplification, the term $e^{{4N_{e} sp\left( {1 - p} \right)}}$ drops from the equation, such that $\phi \left( p \right) \approx Cp^{{4N_{e} v - 1}} \left( {1 - p} \right)^{{4N_{e} v - 1}}$. Let us consider here $C^{ - 1}$ instead of $C$, for ease of notation. Furthermore, because the value of $4N_{e} v$ used here is rather small ($v = 1 \times 10^{ - 8} , N_{e} = 65; 4N_{e} v = 2.6 \times 10^{ - 6}$), we can assume that $4N_{e} v - 1 \approx - 1$. Thus, we obtain that:

$$\begin{aligned} C^{ - 1} & = \mathop \sum \limits_{{p = \frac{1}{2n}}}^{{\frac{2n - 1}{2n}}} \left( {p^{{4N_{e} v - 1}} \left( {1 - p} \right)^{{4N_{e} v - 1}} } \right) \\ & \approx \mathop \sum \limits_{{p = \frac{1}{2n}}}^{{\frac{2n - 1}{2n}}} \left( {p^{ - 1} \left( {1 - p} \right)^{ - 1} } \right) = \mathop \sum \limits_{{p = \frac{1}{2n}}}^{{\frac{2n - 1}{2n}}} \left( {\frac{1}{{p - p^{2} }}} \right). \\ \end{aligned}$$

Now, we rewrite the above, such that the summation is expressed in terms of the “Harmonic series” [7], for which it is known that:

$$\mathop \sum \limits_{m = 1}^{k} \left( {\frac{1}{m}} \right) = \ln \left( k \right) + \frac{1}{2k} + {{\upgamma},}$$

where ${{\upgamma }}$ is the Euler–Mascheroni constant (being close to 0.57721) [8].

With $p = \frac{i}{2n}$, we get:

$$C^{ - 1} \approx \mathop \sum \limits_{{p = \frac{1}{2n}}}^{{\frac{2n - 1}{2n}}} \left( {\frac{1}{{p - p^{2} }}} \right) = 2n\mathop \sum \limits_{i = 1}^{2n - 1} \left( {\frac{1}{{i - \frac{{i^{2} }}{2n}}}} \right).$$

We then note that this series is symmetric, e.g., both $i = 1$ and $i = 2n - 1$ result in $\frac{1}{{1 - \frac{1}{2n}}}$:

$$\mathop \sum \limits_{i = 1}^{2n - 1} \left( {\frac{1}{{i - \frac{{i^{2} }}{2n}}}} \right) = \frac{1}{{1 - \frac{1}{2n}}} + \frac{1}{{2 - \frac{4}{2n}}} + \frac{1}{{3 - \frac{9}{2n}}} + \cdots + \frac{1}{{3 - \frac{9}{2n}}} + \frac{1}{{2 - \frac{4}{2n}}} + \frac{1}{{1 - \frac{1}{2n}}}.$$

For this series, the $i$th element is equal to:

$$\frac{1}{{i - \frac{{i^{2} }}{2n}}} = \frac{{1 - \frac{i}{2n}}}{{i\left( {1 - \frac{i}{2n}} \right)}} + \frac{{\frac{i}{2n}}}{{\frac{i}{2n}\left( {2n - i} \right)}} = \frac{1}{i} + \frac{1}{2n - i}.$$

Such that:

$$\mathop \sum \limits_{i = 1}^{2n - 1} \left( {\frac{1}{{i - \frac{{i^{2} }}{2n}}}} \right) = \mathop \sum \limits_{i = 1}^{2n - 1} \left( {\frac{1}{i}} \right) + \mathop \sum \limits_{i = 1}^{2n - 1} \left( {\frac{1}{2n - i}} \right).$$

The symmetry of the series implies that it is the sum of the harmonic series $\left( {\frac{1}{i}} \right)$ and its reverse $\left( {\frac{1}{2n - i}} \right)$, which are the same, such that:

$$\mathop \sum \limits_{i = 1}^{2n - 1} \left( {\frac{1}{{i - \frac{{i^{2} }}{2n}}}} \right) = 2\mathop \sum \limits_{i = 1}^{2n - 1} \left( {\frac{1}{i}} \right).$$

Thus:

$$\begin{aligned} C^{ - 1} & \approx 2n\mathop \sum \limits_{i = 1}^{2n - 1} \left( {\frac{1}{{i - \frac{{i^{2} }}{2n}}}} \right) \\ & = 4n\mathop \sum \limits_{i = 1}^{2n - 1} \left( {\frac{1}{i}} \right) = 4n\left( {\ln \left( {2n - 1} \right) + \frac{1}{4n - 2} + {{\upgamma }}} \right), \\ \end{aligned}$$

and

$$C \approx \frac{1}{4n}\left( {\ln \left( {2n - 1} \right) + \frac{1}{4n - 2} + {{\upgamma }}} \right)^{ - 1} ,$$

which is denoted as $C^{*}$ in the paper, and holds when $4N_{e} v \approx 0$, and $n$ is not extremely large. In other cases, $C = \left( {\mathop \sum_{{p = \frac{1}{2n}}}^{{\frac{2n - 1}{2n}}} p^{{4N_{e} v - 1}} \left( {1 - p} \right)^{{4N_{e} v - 1}} } \right)^{ - 1}$ should be used.
